# Lack of effects on key cellular parameters of MRC-5 human lung fibroblasts exposed to 370 mT static magnetic field

**DOI:** 10.1038/srep19398

**Published:** 2016-01-14

**Authors:** Stefania Romeo, Anna Sannino, Maria Rosaria Scarfì, Rita Massa, Raffaele d’Angelo, Olga Zeni

**Affiliations:** 1 CNR – Institute for Electromagnetic Sensing of Environment, Via Diocleziano 328, 80124 Naples, Italy; 2 Department of Physics, University of Naples Federico II, CMSA via Cintia, 80126, Napoli, Italy; 3 Italian Workers’ Compensation Authority (INAIL)–Regional Technical Advisory Department Risk and Prevention Assessment (CONTARP) of Campania, via Nuova Poggioreale, 80143 Napoli

## Abstract

The last decades have seen increased interest toward possible adverse effects arising from exposure to intense static magnetic fields. This concern is mainly due to the wider and wider applications of such fields in industry and clinical practice; among them, Magnetic Resonance Imaging (MRI) facilities are the main sources of exposure to static magnetic fields for both general public (patients) and workers. In recent investigations, exposures to static magnetic fields have been demonstrated to elicit, in different cell models, both permanent and transient modifications in cellular endpoints critical for the carcinogenesis process. The World Health Organization has therefore recommended *in vitro* investigations as important research need, to be carried out under strictly controlled exposure conditions. Here we report on the absence of effects on cell viability, reactive oxygen species levels and DNA integrity in MRC-5 human foetal lung fibroblasts exposed to 370 mT magnetic induction level, under different exposure regimens. Exposures have been performed by using an experimental apparatus designed and realized for operating with the static magnetic field generated by permanent magnets, and confined in a magnetic circuit, to allow cell cultures exposure in absence of confounding factors like heating or electric field components.

The issue of biological effects of intense static magnetic fields (SMF) has been increasingly tackled over the last years, due to their wider and wider application in the clinic and research with magnetic resonance imaging (MRI) systems, as well as in transportation systems and in high energy physics research facilities^1^.

Among the cited applications, MRI plants are the main source of exposure to SMF for both general public (patients) and workers. Despite the existence of relevant national and international guidelines and directives limiting the occupational exposure to electromagnetic fields (EMFs)^2^,^3^,^4^, and of a consistent body of literature on MRI safety for patients and workers^5^,^6^, the actual levels of exposure of MRI workers are not unequivocally defined, the exposure depending on several factors, partly related to the characteristics of the workplaces (type of scanner, layout of the MRI facility) and partly to the specific job tasks carried out by the individual worker. This subject has been extensively discussed in a recent review by McRobbie^7^, and a wide measurement campaign of exposure levels has been recently performed by Schaap and co-workers^8^.

These considerations lead to a wide interest for the possible biological effects arising from exposure to moderate (1 mT to 1 T) and strong (>1 T) SMF. In the following, a brief summary of already available results, as from the main studies related to health issues, is presented, to set the scene of the current knowledge so far.

Regarding the human studies, a systematic quantitative analysis on the effects of SMF on cognition, vital signs and sensory perception, revealed significant impairment in visual system and increase of dizziness and vertigo, primarily caused by movement through the SMF or exposure to magnetic field gradients, while vital signs were not affected^9^. Such effects are transient and occur only when the field exceeds certain thresholds^2^,^3^.

A comprehensive review of the bio-effects of static magnetic field on rodent models has been recently presented by Yu and Shang^10^ in which, potentially therapeutic benefits, in the case of moderate intensity, as well as adverse effects, in the case of acute strong SMFs, have been evidenced.

Concerning *in vitro* laboratory investigations, available studies have been carried out on different cell models, by adopting a wide range of exposure protocols in terms of magnetic induction levels and exposure duration and timing (continuous or intermittent), and by addressing different biological endpoints. In particular, primary human cells as well as cell lines were mainly employed, both healthy and cancer models, but also murine cells were considered. For SMF exposures, either low (60 μT to 40 mT), moderate (100 to 700 mT) or high (1 to 10 T) induction levels were adopted, and exposure duration ranged from few minutes to hours or several days. The main biological endpoints considered were oxidative stress, gene expression and genotoxicity, cell viability and growth^11^. From the most recent investigations, exposure to SMF resulted in alterations of the expression of specific genes, with dependence of such effect on exposure duration and field gradients. Genotoxic effects have been reported under certain conditions, although in most cases they were repaired and not permanent. Contrasting results have been reported on cell viability and growth, as well as on oxidative stress with some evidences suggesting possible interference of the field with the cell redox status^12^,^13^,^14^,^15^.

Overall, despite the fair number of studies published so far, adequate data strongly supporting an appropriate health risk evaluation of SMFs are still lacking, and the World Health Organization (WHO) recommended that authorities should increase the research effort on the study of health effects of SMF^1^.

In this study, we present experimental results obtained in human foetal lung fibroblast cells (MRC-5 cell line), exposed to 370 mT magnetic induction under different exposure timing. To this aim, the design and characterization of an experimental apparatus, which allows exposure of cell cultures to SMF under controlled conditions, is also reported. The rationale behind the definition of both magnetic induction level and exposure protocol was to include exposure conditions which likely occur in the framework of MRI clinical procedures.

## Results

### Intermittent SMF exposure did not affect cell viability and intracellular ROS levels

**Figure 1:**
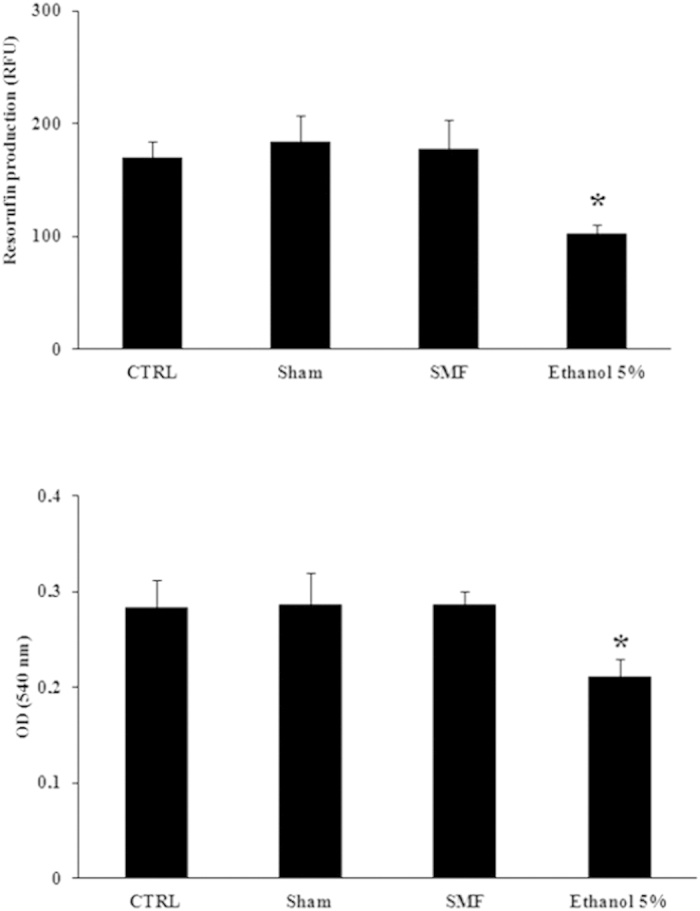
Absence of effect on cell viability of MRC-5 cells exposed to 370 mT magnetic induction level. Intermittent (1 h/day for 4 days) SMF exposure did not affect cell viability in the resazurin (top) and neutral red (bottom) assays. Data are presented as mean ± SD of six independent experiments. Results of ethanol treatment (5%, 15 min) are also presented as positive control. *P < 0.05, one-way ANOVA multiple –comparison followed by Bonferroni test.

Figure 1 presents the results on viability of MRC-5 cells, in terms of metabolic activity (top) and membrane integrity (bottom), as obtained, from the resazurin assay and neutral red assay, respectively. In six independent experiments, control (CTRL), sham exposed (Sham), and SMF-exposed cells, exhibited the same resorufin production and the same ability to take up neutral red. As expected, in both assays, a reduction in cell viability was recorded in ethanol treated cells (5% ethanol for 15 min, positive control) as compared to the other treatments (P < 0.05).

**Figure 2:**
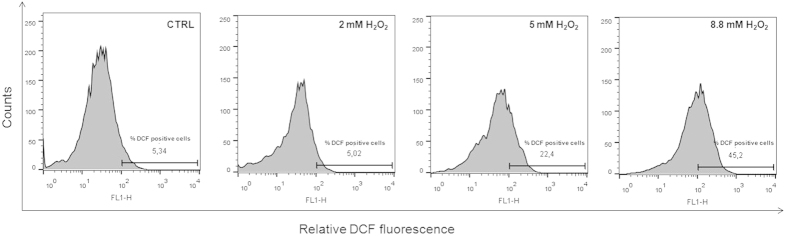
Increased ROS levels in MRC 5 cells treated with hydrogen peroxide (H_2_O_2_). Treatment of 30 min with increasing concentrations of H_2_O_2_ resulted in increased ROS levels. The percentage of DCF positive cells for 2, 5, 8.8 mM H_2_O_2_ final concentration is presented, as from the DCF fluorescence histograms analyzed by the Flow Jo analysis program. The % of DCF-positive cells was quantified considering a threshold fluorescence level of 10^2^ (a.u.), set on the basis of the background fluorescence in the control cell population.

In six independent experiments, intracellular reactive oxygen species (ROS) levels also resulted unaffected as shown in Table 1, where the percentage of DCF positive cells in CTRL, Sham, SMF exposed samples is presented. Increased ROS levels were, instead, detected in MRC-5 cells after 30 min treatment with increasing concentration of H_2_O_2_, demonstrating the sensitivity of the applied method. As a matter of fact, in the DCF fluorescence histograms presented in Fig. 2, the percentage of DCF positive cells increased upon increasing H_2_O_2_ final concentration, the minimum effective being 5 mM.

### Continuous SMF exposure for 24 h did not alter DNA integrity

In order to verify whether SMF exposure is capable of inducing primary DNA damage in MRC-5 cells, 24 h continuous exposure to 370 mT magnetic induction level were tested. The results obtained in the comet assay are presented in Table 2, where tail DNA %, tail moment (a.u.), tail length (μm), as a measure of DNA migration, and the number of hedgehogs, as a measure of early apoptotic events, are presented. In three independent experiments, CTRL, Sham, and SMF-exposed cells, exhibited no statistically significant alteration in the comet parameters analyzed, and a comparable number of hedgehogs. Treatments with H_2_O_2_ (25 μM for 10 min) induced a noticeable increase in the comet parameters and in the number of hedgehogs (p < 0.05). None of the treatments caused a significant decrease in cell viability, in the trypan blue dye exclusion assay, which was above 90% in all cases (data not shown).

## Discussion and Conclusion

In the evaluation of the potential biological effects that could arise from exposure to SMF, well controlled *in vitro* exposures are required. As a matter of fact, the peer-reviewed literature on *in vitro* studies is far to be conclusive, and does not provide any foundation or support to *in vivo* and epidemiological studies for a proper risk evaluation. In very recent literature, great attention has been devoted to study the effects on selected cellular endpoints related to cancer development, in different cell models. Gene expression and genotoxicity^15^,^16^,^17^,^18^,^19^,^20^, oxidative stress^21^,^22^,^23^,^24^, cell growth, differentiation and viability^16^,^22^,^25^,^26^,^27^,^28^,^29^ have been investigated. In a consistent portion of the available studies, SMF exposure induced effects in the cellular endpoints that, in some cases, were transient. Some evidences have also suggested that many cell processes can be influenced by combined application of SMF and drugs^20^,^30^,^31^. Thus, investigations aimed at clarifying the discrepancies, and verifying the evolution of such transient modifications are of crucial importance.

Here we presented the design and characterization of an *in vitro* device based on Neodymium-Fe-Boron permanent magnets. The procedures described here could represent a tool for researchers who intend to expose cell cultures to SMF in absence of confounding factors like heating or electric field component, that could affect the results in the case of electromagnets based exposure devices. Moreover, the accurate description of such procedures will allow replication of the experiments in independent laboratories, and thus account for one of the most critical points in bioelectromagnetic research. As a matter of fact, one of the sources of failure of replication studies aimed to reproduce already published investigations reporting effects, is the absence of detailed description of exposure set ups and conditions. Therefore, it is widely recognized that one of the main requirements for *in vitro* studies addressing biological effects of EMFs exposure is the employment of detailed procedures for the choice, design and set up of exposure system, accurate determination of the electric and/or magnetic fields in the exposed samples, in compliance with physiological conditions for cell cultures. Moreover, the presence of sham-exposed controls is also critical in these type of studies, in order to ascertain that eventual observed effects can be ascribed to the EMF exposure and not to environmental conditions inside the exposure chamber^14^.

By using such a device, human foetal lung fibroblasts (MRC-5) were subjected to SMF exposures at induction level of 370 mT, given as on/off cycles of 1 h/day for 4 consecutive days, for which a more complex biological response with respect to continuous exposures is expected. In this case, possible effects on cell viability and ROS formation were evaluated. As a matter of fact, cell viability is one of the first response to be investigated under different stress conditions, and can be regarded as a measure of permanent damage. On the other hand, it has been hypothesized that SMF can increase the activity, concentration and lifetime of paramagnetic free radicals, which may cause oxidative stress, genetic mutation and/or apoptosis and alteration of cell viability^31^. Further, in order to provide some mechanistic insights on the interaction between SMF and human cells, possible effects on DNA integrity have been investigated after 24 h continuous exposures. As a matter of fact, in the alkaline comet assay, effects at the level of DNA molecule that could be repaired, and thus not necessarily translated in detectable effects on cellular response, can be captured.

In our experimental conditions, neither permanent effects nor transient modifications have been elicited by SMF exposures. As a matter of fact, neither intermittent nor 24 h continuous exposures to 370 mT magnetic induction level were able to alter viability of MRC-5 cells. Moreover, intermittent and continuous exposures were also unable to evoke effects in the form of ROS formation and primary DNA damage, respectively. ROS levels and DNA migration pattern, as from the intracellular DCF fluorescence measurement and comet assay respectively, capture cellular modifications that could be repaired by intracellular repair mechanisms, and not necessarily translated in unbalanced oxidative metabolism and permanent DNA damage. These two measurements are thus able to detect also transient effects. Furthermore, our experiments, taking advantages from the alkaline comet assay, also allowed to gain some clues about the absence of early apoptotic events under SMF exposure, although effects on apoptosis cannot be ruled out, since the validity of such measurement in detecting apoptosis is still under debate^32^.

Some recent studies reported transient increase in ROS formation in different cell types after exposure to either moderate (35 to 300 mT)^22^,^23^, or very strong (8.5 T) SMF^24^, with exposure duration ranging from few hours up to 24 h. Some investigations also reported permanent increase of ROS with weak SMF (5 mT) with exposure duration ranging from few minutes up to 2 h^21^. Therefore, no indications on possible dose-response effects or of dependence on exposure parameters can be extracted by such positive findings. Both positive and negative findings have been reported on cell viability and DNA damage, under not easily comparable exposure protocols^31^. Our study is in partial contrast with those cited above, and it is not possible, on the basis of the current knowledge, to ascribe such discrepancies to either exposure parameters or to cell type. Nevertheless, also negative findings provide useful information in the framework of the evaluation of possible health effects arising from human exposure to SMF, which has increased over the last years due to their large use in industrial applications and in the clinical practice. Among such applications, MRI facilities are the most widespread sources of exposure, involving both patients and workers, although with different modalities.

In the proximity of MRI systems, different types of EMFs, from SMF, to rapidly changing gradient magnetic fields and radiofrequency fields (10–100 MHz), are simultaneously present. Among these different EMFs, the SMF from MRI scanner is always turned on, and therefore both patients and different categories of workers (technicians, medical doctors, nurses, cleaning personnel) can be exposed during the clinical procedures. The magnetic induction level (B) generated by MRI systems typically range from 0.2 T to about 3 T in the bore, where the patient lies during the diagnostic examination, it extends beyond the confines of the scanner bore, and is reduced with the distance from the scanner, generating a spatial gradient of magnetic field (stray field). The occupational exposure takes place while attending patients before and after examination, during particular procedures, when the patient needs assistance during the examination, and also while operating the scanner’s console^33^. Health care staff can be in average exposed to B levels of up to 500 mT^8^. Moreover, the actual trend is toward the development of MRI systems operating with higher and higher field strengths, which allow to increase the performance of the diagnostic system by improving the signal-to-noise ratio, the sensitivity to soft tissues, and the spatial resolution of the images^34^. According to the analysis carried out by Schaap and co-workers^8^, the exposure levels to the static stray field in a 3 T MRI facility can be approximately 1.5 times higher than in a 1.5 T MRI, while switching from 3 T to 7 T would result in exposure levels almost 10 times higher.

The magnetic induction level of 370 mT adopted in this study can be traced to a real case of exposure, by considering the numerical analysis and the measurements reported by Crozier and co-workers^35^ and Fuentes and co-workers^36^. In particular, exposure to 370 mT can occur in MRI plants with either 1.5 T, 4 T or 7 T magnets, at approximate distances from the bore of 1 m, 1.5 m and 2 m, respectively. It is therefore likely that such exposure occurs during routine procedures, like patient assistance and preparation, and involves the professional categories, both technical and medical staff, that are allowed to enter the MRI suite. Further, the intermittent exposure protocol (1 h/day for 4 days) here adopted, represents one of many possible scenarios of occupational exposure, that are strictly related to the specific activities carried out and to working procedures.

In conclusion, here we report on the absence of effects on selected cellular endpoints in MRC-5 cells exposed to a moderate intensity, but realistic for occupational exposure, SMF, by using an ad hoc devised system which allows cell cultures exposure and sham-exposures under strictly controlled conditions.

Research along the lines adopted in our investigations, across different cell models, warrants further and extensive consideration in order to shed light on possible interactions between SMF and cellular processes. Such a research will also help in establishing guidelines for occupational and patient exposures to static magnetic fields in MRI suites.

## Materials & Methods

### Design, realization and characterization of the exposure device

An exposure device was designed, realized and characterized in order to expose cells to SMF under strictly controlled electromagnetic and environmental conditions. In particular, the design was driven by the following working hypotheses: 1) possibility to host both the exposure and the sham/exposure devices inside a cell culture incubator, in order to perform long exposures; 2) device based on small permanent magnets, in such a way to avoid confounding factors like electric field components or heating that could arise in the case of electromagnets; 3) possibility to confine the magnetic field lines generated by the magnets, in such a way to maximize the magnetic induction level and field uniformity in the sample area.

**Figure 3:**
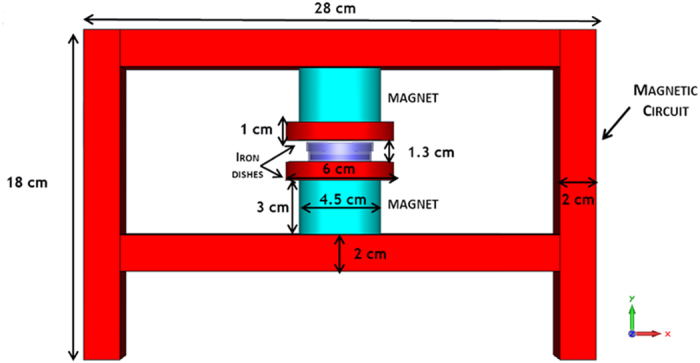
The configuration of the exposure device. Layout and dimensions of the static magnetic field exposure device. The cell culture Petri dish, hosted in between the two iron dishes, is also shown.

The design has been performed by using the CST EM Studio (Darmstadt, Germany) software: magneto-static simulations have been run in order to pursue the best system configuration, allowing to obtain a considerable magnetic induction level for sample exposure, without increasing too much the dimensions of the whole system. The exposure device has been configured as a magnetic circuit able to confine the magnetic field generated by a couple of permanent magnets, placed in the centre of the circuit and at a suitable distance between each other, in such a way to allow the insertion of the sample. For the choice of the permanent magnets, two neodymium-iron-boron (Ne-Fe-B) elements have been considered, selected from those available on the market on the basis of physical and geometrical characteristics. The magnetic circuit has been simulated as an iron structure, while two dishes of the same material, placed between the magnets, have been considered which allow to increase the field uniformity in the sample position. The magneto-static simulations have been configured by setting up a permanent magnet source with axial magnetization, as indicated by the magnets manufacturer (Webcraft GMBH, supermagnete.com). The final configuration of the exposure device is shown in Fig. 3. Cylindrical magnets, 30 mm high, 45 mm diameter, magnetic remanence of 1.32–1.37 T, have been preferred to rectangular ones which would have required larger dimensions, and consequently larger weight of the structure. The magnetic circuit is made by iron plates, 2 cm thick, and the overall structure is 18 cm high, 28 cm wide and 20 cm deep. The two iron dishes placed in the center (1 cm thick, 6 cm in diameter) are distant 1.3 cm between each other, allowing the insertion of a Petri dish in between.

**Figure 4:**
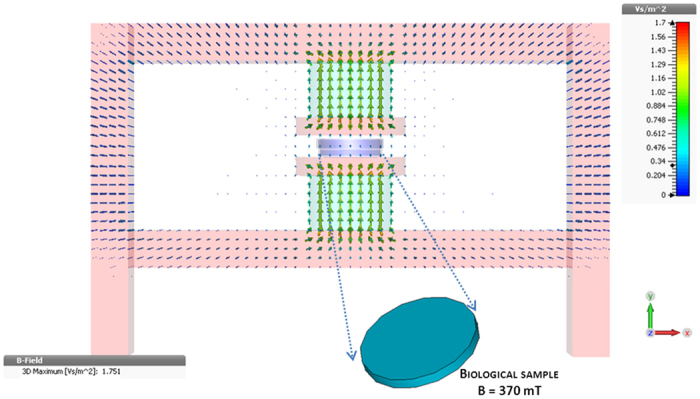
The magnetic induction level in the exposure device. Distribution of the magnetic induction in the sample and through the magnetic circuit (the color bar is expressed in Vs/m^2^, where 1 Vs/m^2^ = 1 T). The homogenous magnetic induction (370 mT) induced in the biological sample is shown in the inset.

The distribution of the magnetic field in the exposure system, as from magneto-static simulations, is shown in Fig. 4. The field is perpendicular to the sample and is perfectly confined by the magnetic circuit. The average magnetic induction level in the sample area is of 370 mT (±0.09%).

**Figure 5:**
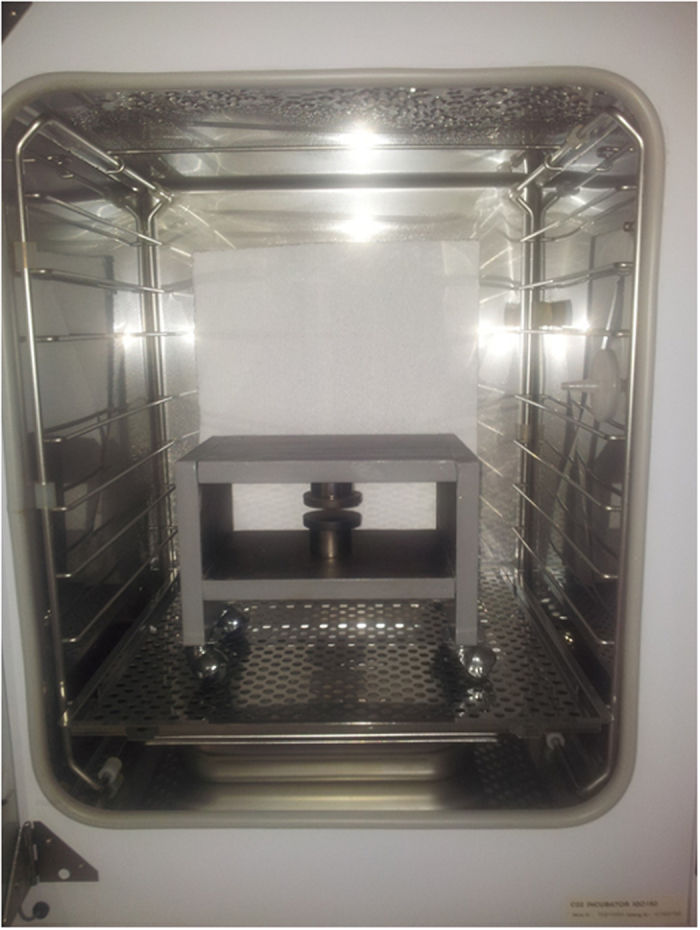
The static magnetic field exposure device. It was realized according to the design configuration, and placed in a standard cell culture incubator to allow cell culture exposure.

The system was then realized by assembling together the iron plates and positioning the magnets and the dishes in the center of the structure. Since the final weight of the system resulted in 22 kg, it has been equipped with wheels to improve its portability (Fig. 5).

After setting up the system, it has been characterized by mapping the magnetic induction levels in the sample area, at different positions and heights between the magnets, by means of a Hall gauss-meter (F.W. Bell, model 4048, accuracy ±2% of reading) fixed on a manual micro-positioner. The measurements resulted in an average value of 366 mT (±0.27%). The exposure device has been hosted inside a standard cell culture incubator (model 311, Forma Scientific, Freehold, NJ, USA) (Fig. 5). An identical structure, with plastic cylinders instead of the magnets, was set up to allow sham-exposure and hosted in a separate cell culture incubator.

### Chemicals and reagents

DMEM medium, Foetal Bovine Serum (FBS), L-glutamine, penicillin-streptomycin, trypan blue and trypsin were from Biowhittaker (Verviers, Belgium), TRIS/HCl was from Applichem (Darmstadt, Germany); resazurin, neutral red, triton X-100, dithiothreitol (DTT), propidium iodide (PI), 2′7′-Dichlorofluorescin diacetate (DCFH-DA), hydrogen peroxide (H_2_O_2_) were from SIGMA (St. Louis, MO, USA); sodium chloride (NaCl), sodium citrate, sodium acetate, acetic acid and ethanol, were from JT Baker (Deventer, Holland). Low-melting point agarose, normal-melting agarose, ethidium bromide were from Bio-Rad Laboratories (Munich, Germany).

### Cell culture and maintenance

Human foetal lung fibroblast cell line (MRC-5) was purchased from the National Institute for Cancer Research (Genova, Italy). Cells were cultured in Dulbecco’s modified Eagle medium (DMEM) with 10% heat-inactivated foetal bovine serum, 2 mM L-glutamine, 100 U/ml penicillin, and 100 mg/ml streptomycin, at 37 °C in an atmosphere of 95% air and 5% CO_2_. For consistency and reproducibility, cell cultures were routinely maintained as monolayer by sub-culturing twice per week by trypsinization. For the experiments, different cell seeding, according to the biological endpoint under examination, was performed in 3 ml complete medium in 35-mm-diameter Petri dishes (Corning, NY).

### Experimental procedures

Different experimental procedures were adopted, based on the biological endpoints under examination, and described in the following.

To assess cell viability and ROS formation, 4 × 10^4^ cells were seeded in 3 ml complete medium. After 72 h of cell growth, culture medium was replaced with fresh medium, and cell cultures were exposed/sham exposed to 370 mT SMF for 1 h/day for 4 consecutive days. Cells were harvested 24 h later.

To assess DNA integrity, 7 × 10^4^ cells were seeded in 3 ml complete medium. After 72 h of growth, culture medium was replaced with fresh medium, and cell cultures were exposed/sham exposed to 370 mT SMF for 24 h. Immediately after exposure, cells were processed for the alkaline comet assay. At the same time, cell viability by trypan blue exclusion dye assay was also recorded.

**Figure 6:**
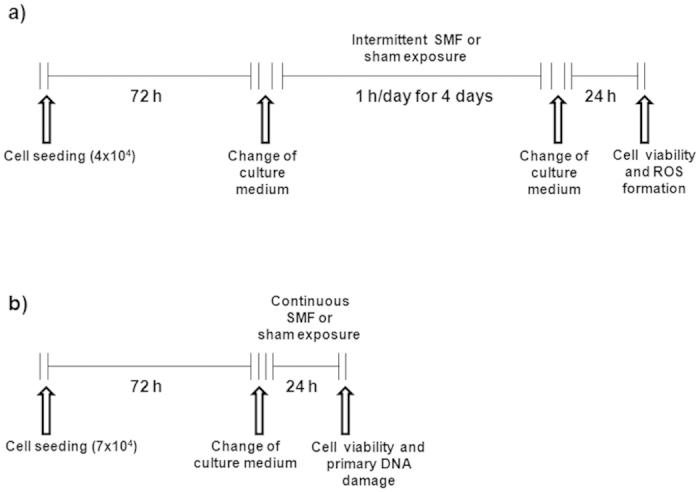
A schematic representation of the experimental procedures. To assess the effects of SMF exposure in MRC-5 cells, different experimental procedures were adopted based on the biological endpoints under examination. They are described here for intermittent (**a**) and continuous (**b**) exposures to SMF.

For the sake of clarity, a schematic representation of the two procedures is presented in Fig. 6.

For each endpoint, the groups of samples were: sham exposed cells (Sham), SMF exposed cells (SMF), control cells, i.e. cells kept in standard cell culture incubator (CTRL) and positive control cells i.e. cells subjected to different treatments, able to evoke damage in MRC-5 cells in the specific biological assay.

After any exposure/treatment, cell samples were coded in order to keep the treatment groups unknown to the researcher involved in the analysis. Codes were broken only at the end of data analysis.

### Measurement of cell viability

Resazurin and neutral red assays were employed, which assess the metabolic activity and the plasma and/or lysosomal membrane integrity, respectively, as a measure of cell viability.

In the resazurin assay, the non fluorescent compound, resazurin, is used, which is reduced to highly fluorescent resorufin in the growth medium by cell activity, and a direct correlation exists between the reduction of resazurin and the metabolic activity of living cells^37^,^38^. After treatments, cell monolayers were incubated for 20 min at 37 °C with 10 μg/mL resazurin in PBS (assay medium). Resorufin production was analysed in the assay medium with a fluorometer (Perkin-Elmer, LS50B, Perkin-Elmer Instruments, Norwalk, CT) at an excitation and emission wavelength of 530 and 590 nm, respectively, and expressed as Relative Fluorescence Unit (RFU).

The neutral red assay examines the ability of cells to incorporate the water-soluble dye, neutral red, into lysosomes in an energy requiring process. Treatments damaging plasma and/or lysosomal membranes, or interfering with the normal energy-requiring endocytosis process, will decrease the ability of cells to take up neutral red^39^. After treatments, cell monolayers were treated with 0.066% (v/v final concentration) neutral red for 3 h, washed in PBS, and after trypsinization, cell suspensions were treated with cold lysis buffer prepared with 50 mM TRIS/HCl, pH 7.4, 150 mM NaCl, 5 mM DTT, 1% Triton X 100, containing 1% acetic acid and 50% absolute ethanol. The optical density of lysed cells at 540 nm (OD 540 nm) was measured (Microplate Reader 680, Bio-Rad Laboratories, Hercules, CA, USA), and was used as an estimation of cell viability.

In both assays, ethanol treatment (5% for 15 min) served as positive control, and six independent experiments were carried out.

### Measurement of intracellular ROS levels

The fluorescent probe 2′,7′-dichlorofluorescin diacetate (DCFH-DA) was used, which is a non-polar compound that easily passes the cell membrane and is hydrolysed by intracellular esterases to the non-fluorescent polar derivative, DCFH. In the presence of ROS, DCFH is oxidised to fluorescent dichlorofluorescein (DCF)^40^.

The assay was carried out as follows: after treatments, cell monolayers were loaded (20 min at 37 °C) in absolute DMEM medium w/o serum containing 5 μM final concentration DCFH-DA. After washing twice in cold PBS, cell monolayers were trypsinized, and DCF fluorescence in the cell suspensions was measured by a flow cytometer (FACScalibur, Becton & Dickinson, San Jose, CA) equipped with a 488 nm argon laser. For each sample, 10000 events were acquired using CELL QUEST software, and the raw data were quantitatively analyzed using the FlowJo analysis program (TreeStar, OR, USA).

Treatments of 30 min with increasing concentrations of H_2_O_2_were carried out to trigger ROS formation in MRC-5 cells and test the sensitivity of the method (positive control). Six independent experiments were carried out, and the results were reported as the percentage of DCF positive cells, i.e. cells expressing DCF fluorescence levels above a threshold value which was set on the basis of the background DCF fluorescence in the control population.

### Measurement of DNA integrity

The alkaline version of comet assay was performed according to the method developed by Singh and co-workers^41^ with minor modifications. Optimum lysis, unwinding and electrophoresis conditions were determined, in preliminary experiments, to obtain detectable DNA migration in control cells^42^, and a subsequent higher sensitivity^43^ of the method in our hand.

The method is basically as follows. After treatments, cells were collected by trypsinization, and cell viability was assessed using the trypan blue exclusion method. For each treatment, 2 slides were set up by suspending aliquots of 5 × 10^4^ viable cells in 100 ml low-melting point agarose (0.6% w/v), sandwiched between a lower layer of 1.5% normal-melting agarose at 37 °C and an upper layer of low melting point agarose (1.5% w/v) on microscope slides. The slides were then immersed for 60 min in a freshly prepared cold lysing solution prepared with 2.5 M NaCl, 100 mM Na_2_ EDTA, 10 mM Tris, pH 10, with 1% Triton X-100 and 10% dimethyl sulphoxide at 4 °C added just before use. At the end of lysis treatment, slides were drained and placed in a horizontal gel electrophoresis tank with freshly prepared alkaline electrophoresis buffer (300 mM NaOH, 1 mM Na_2_ EDTA, pH 13) and left in the solution for 20 min at 4 °C to allow the equilibration and DNA unwinding to express alkali labile damage. Using the same buffer, electrophoresis was carried out at 4 °C for 40 min at 30 V by using an Amersham Pharmacia Biotech power supply (Uppsala, Sweden) and adjusting the current to 300 mA by modulating the buffer level. Then, slides were rinsed three times with Tris (400 mM, pH 7.5), rinsed again in distilled water, and air-dried in the dark. All the steps described were conducted under dimmed light to prevent additional DNA damage. Immediately before analysis, slides were stained with 12 μg/ml ethidium bromide. For each treatment, images of 500 randomly selected nuclei (250 from each duplicate slide) were analyzed to detect small but significant effects^44^, by using a computerized image analysis system (Delta Sistemi, Rome, Italy) fitted with a Leica DMBL fluorescence microscope (Leica Microsystems, Mannheim, Germany) at 200 X magnification. DNA integrity was evaluated by calculating the percentage of migrated DNA, tail length, and tail moment^42^.

On the same slides, the number of “hedgehog” comets, characterized by almost all DNA in the tail and very small head, were recorded to have a gross estimation of possible early apoptosis events.

Treatment with H_2_O_2_ (25 μM for 10 min) was employed to evoke DNA damage in MRC-5 cells, and served as positive control. Three independent experiments were carried out.

### Statistical analysis

Statistical comparisons among the groups of samples (Sham, SMF, CTRL and positive control) were conducted with one-way ANalysis Of VAriance (ANOVA) for multiple comparison at the 95% confidence level. A *post hoc* Bonferroni test was performed, and *P* < 0.05 was considered statistically significant.

## Additional Information

**How to cite this article**: Romeo, S. *et al.* Lack of effects on key cellular parameters of MRC-5 human lung fibroblasts exposed to 370 mT static magnetic field. *Sci. Rep.* **6**, 19398; doi: 10.1038/srep19398 (2016).

## Figures and Tables

**Table 1:** Intracellular ROS levels of MRC-5 cells subjected to intermittent SMF exposure at 370 mT magnetic induction level.

	Percentage of DCF positive cells		
Experiment	CTRL	Sham	SMF
1	5.34	5.08	7.13
2	7.47	2.72	6.15
3	5.91	1.11	2.15
4	7.46	5.93	1.4
5	2.23	2.92	3.28
6	2.46	1.73	3.8
**Mean** ± **SD**	**5.15** ± **2.33**	**3.25** ± **1.89**	**3.99** ± **2.24**

The percentage of DCF positive cells in incubator control (CTRL), in sham exposed (Sham) and in SMF exposed (SMF) cells of six independent experiments is reported.

**Table 2:** DNA migration pattern of MRC-5 cells in the alkaline comet assay after 24 h SMF continuous exposure/sham exposure at 370 mT magnetic induction level.

Experiment	Treatment	Migrated DNA (%)	Tail length (microns)	Tail moment (arbitrary units)	Apoptotic nuclei
1	**CTRL**	3.00	6.75	0.93	6.00
	**Sham**	2.85	6.20	0.80	2.00
	**SMF**	2.97	6.08	0.79	2.00
	**H**_**2**_**O**_**2**_ **25 μM**	10.07	23.00	4.28	11.00
2	**CTRL**	2.43	7.04	0.91	1.00
	**Sham**	2.76	7.00	0.92	1.00
	**SMF**	2.64	6.93	0.91	1.00
	**H**_**2**_**O**_**2**_ **25 μM**	9.41	30.63	5.76	5.00
3	**CTRL**	2.92	5.40	0.79	6.00
	**Sham**	4.34	7.70	1.45	5.00
	**SMF**	3.19	5.86	0.90	6.00
	**H**_**2**_**O**_**2**_ **25 μM**	7.54	16.45	2.51	11.00
**Mean** ± **SD**	**CTRL**	**2.78** ± **0.30**	**6.40** ± **0.88**	**0.87** ± **0.07**	**4.33** ± **2.88**
	**Sham**	**3.32** ± **0.88**	**6.97** ± **0.75**	**1.05** ± **0.34**	**2.67** ± **2.08**
	**SMF**	**2.93** ± **0.27**	**6.29** ± **0.57**	**0.86** ± **0.06**	**3.00** ± **2.64**
	**H**_**2**_**O**_**2**_ **25 μM**	**9.01** ± 1.31*	**23.36** ± 7.10*	**4.18** ± 1.63*	**9.00** ± 3.46*

Results of H_2_O_2_ treatment (25 μM, 10 min) are also presented as positive control. *P < 0.05, one-way ANOVA multiple –comparison followed by Bonferroni test.
